# New understanding of electrical activity brought by surface potential of cardiomyocytes

**DOI:** 10.1038/s41598-021-86138-w

**Published:** 2021-03-23

**Authors:** Ying Zhou, Yanfei Hao, Pei Sun, Guang Li, Mengqi Dong, Xuehui Fan, Xiuyun He

**Affiliations:** 1 The Eighth Medical Center, Chinese People′s Liberation Army General Hospital, Beijing, 100091 People’s Republic of China; 2 Institute of Cardiovascular Research, Southwest Medical University, Luzhou, 646000 People’s Republic of China

**Keywords:** Physiology, Cardiology, Chemistry

## Abstract

Aiming at the problem encountered in the previous research: during the electrical activity of cardiomyocytes, the influent ions do not seem to be directly derived from the extracellular fluid. We chose to cut in from the colloidal properties of the cells, follow the basic principles of physical chemistry, and establish hypotheses along the derivation of the structural characteristics of cardiomyocytes. Through the surface ion adsorption experiment and patch clamp experiment of living cells, under the condition of sequentially reducing the concentration of Na^+^ in the extracellular fluid, we observed the exchange and diffusion of adsorbed ions on the cell surface; the changes of inflow *I*_Na_, *I*_Ca-L_ and action potential; and correlation between results. The results showed that the hypothesis is true. The observed parameter changes were consistent with the fact that during depolarization of cardiomyocytes, the ions of influx were derived from the inference of adsorbed ions on the cell surface; at the same time, it also provided an objective and realistic explanation for the generation of electrocardiogram.

## Introduction

How does the myocardial cell (heart) generate surface potential difference and ECG during electrical activity? That has been bothering us. Depending on the rate and method of ion diffusion in the solution^1^, and the method of collecting the electrocardiograph signal^2^, based on the volume of the cell and the surface area of the membrane^3^, in the open environment (flowing liquid), the inflow of ions cannot detect the potential difference on the cell surface^4^. So, how is the surface potential difference of the cardiomyocytes (that is, after the ion adsorption layer on the cell surface is separated, the potential difference between the cell surface and the extracellular fluid) that continues the entire electrical activity process detected (and the size of depolarization potential and repolarization potential are different)?

The membrane components of cardiomyocytes are mainly negatively charged, and the surface shows a negative charge^5^, and its electrical activity is triggered by an external electric field or a change in surface potential^6^. This indicates the colloidal properties of cardiomyocytes and the importance of colloidal properties in the process of electrical activity. Therefore, the research on the charged components of the myocardial cell membrane, the surface potential, the adsorbed ions on the membrane surface, the ion adsorption layer, etc., the action in the process of electrical activity, and the exchange and diffusion of adsorbed ions in the process of electrical activity seems particularly important. Over the years, we have made more detailed explorations along this line. It is recognized that the composition of the surface potential (that is, the potential difference between the surface of a charged cell and the inside of the extracellular fluid) of cardiomyocytes includes both membrane charges (fixed charges) and intracellular charges (active charges). Membrane charge includes: the negative charge carried by phospholipid membrane (phosphatidylserine, etc.), membrane proteins and surface glycoconjugates (sialic acid, etc.); intracellular charges, that is, transmembrane potential (the potential difference between the inner and outer fluid of the cell). The cell surface potential of multiple components, collecting the differences in space and distance (different layers), as well as the characteristics of dynamic correlation with electrical activity, which forms a surface ion adsorption layer with complex structure and changes. At the same time, the electrical activity of the cell has the following characteristics: 1. In the surface potential, the change of each component will cause the size and shape of the ion adsorption layer on the surface to change. The change of the transmembrane potential is affected by the influx of positive ions. When the transmembrane potential increases (the negative value becomes smaller), it directly causes the surface ion adsorption layer to become smaller (the amount of adsorbed ions decreases) and the shape changes, causes the surface of the cell to release adsorbed positive ions into the suspension (extracellular fluid). During the entire depolarization process, the outward positive ion flow on the cell surface is formed. 2. The initiation of the electrical activity of the cardiomyocytes in vivo is the same as the cell electrophoresis experiment process, which is triggered by the external electric field and completed in the external electric field (the external electric field of the ventricular myocytes in the body comes from the interaction between cells in the microspace). That is, during the electrical activity, the cell surface is continuously negatively charged. In this way, we seem to have found the logical basis and origin of the formation of electrocardiogram (there is a potential difference on the cell surface).

However, if so: in the external electric field, the surface of the cell loses adsorbed ions and is charged (electric double layer separation), and after the ion channel is open, the inward positive ions cannot come from the extracellular fluid; at the same time, the inward flow of positive ions increases the transmembrane potential, forming a continuous outward with positive ion flow, the positive ions in the extracellular fluid are also impossible to enter the cell. That is to say, in the process of depolarization, the inflow of positive ions is unlikely to originate from extracellular fluid. The cells are in a relatively "isolated space" in the suspension, and at the same time there is the influence of the electrochemical potential inside and outside the cells, inward ions can only come from the adsorbed ions on the cell surface. Of course, this is also consistent with the fact that after depolarization the surface potential difference of the cardiomyocytes (heart) rises^7^.

This is an important question about the origin of theoretical understanding. In order to figure out the source of inflow ions in the electrical activity of cardiomyocytes, and then find the correct answers to related questions, this research starts from how to show the difference between the two ion diffusions in the suspension and the membrane surface adsorption, based on the colloidal properties of biological cells^8^ and the rules of biofilm ion adsorption, exchange and diffusion^9^. And we establish a hypothesis: it is assumed that in the electrical activity of cardiomyocytes, the influx ions come from the adsorbed ions on its surface. At the same time, the rat ventricular myocytes were selected to reduce the Na^+^ concentration in the extracellular fluid and changed the composition ratio between ions in a large span. A complete live cell surface adsorption experiment and patch-clamp technique were used to detect changes in the adsorbed ions on the cell surface and changes in inflow *I*_Na_, *I*_Ca-L_ and action potential (AP) during the experiment, to obtain the true source of inward ions in the electrical activity of cardiomyocytes.

## Results

The surface structure of the isolated and in vivo cardiomyocytes is slightly different. The separation will enzymatically remove some charged ingredients on the cell surface, mainly surface glycoconjugates, which will slightly reduce the number of adsorbed ions on the cell surface. However, the removed quantity is only a small part of the surface glycoconjugates of the cell, and has little influence on related parameters, and generally does not affect qualitative research. In the preparation of the experimental solution, we reduced the concentration of NaCl and added glucose to maintain the osmotic pressure of the cells. Glucose is non-electrolyte and does not affect the adsorption and exchange of ions on the cell surface; its entry into cardiomyocytes depends on the glucose transporter on the cell membrane. The number of transporters on the membrane determines the speed of glucose transport, and for isolated cardiomyocytes, it is mainly regulated by insulin, etc^10,11^. And for acute high glucose, it will prolong the AP duration of cardiomyocytes of healthy rats (related to outflow K^+^). We refer to Warda's research^12^, utilize the difference of onset time of positive ions and glucose, shorten the suspension time of the cells in the high glucose solution, and avoid adding glucose transporter regulators to the experimental solution. In the patch clamp experiment, a method of pairing and perfusion between the control group and the experimental group was adopted; the records of the experimental group were controlled to be completed within 5 min, which means smaller impact on the results.

### Changes in adsorbed ions on cell surface

**Figure 1:**
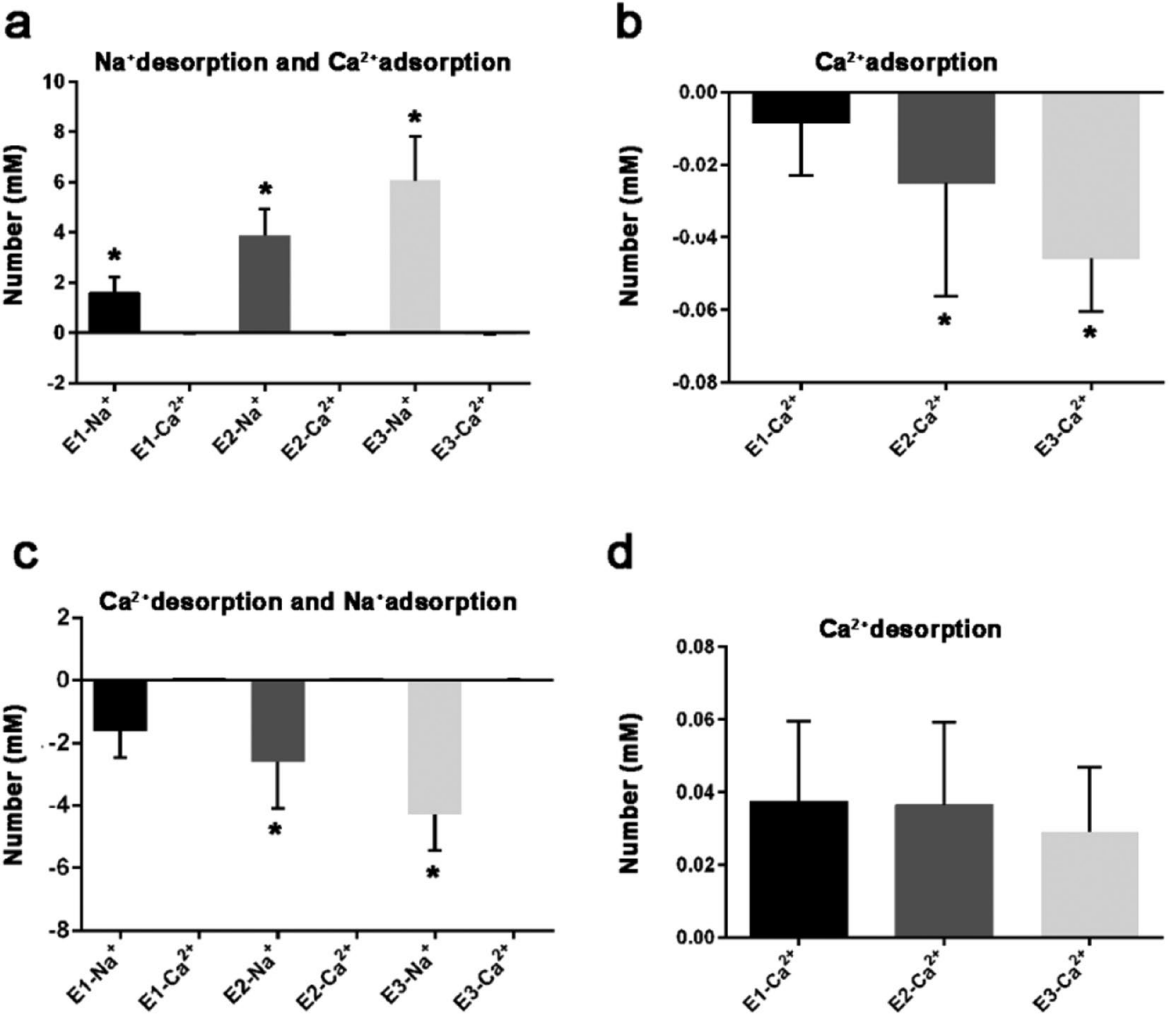
Impact of Na^+^ concentration change in extracellular fluid on the adsorbed Ca^2+^ and Na^+^ of cardiomyocytes surface. In the Ca^2+^ adsorption experiment, the number of Ca^2+^ adsorbed was compared with the number of Na^+^ desorbed (**a**). The number of Ca^2+^ was adsorbed (**b**). In the Na^+^ adsorption experiment, the number of Na^+^ adsorbed was compared with the number of Ca^2+^ desorbed (**c**). The number of Ca^2+^ was desorption (**d**). (E-1, E-2 and E-3 were 105 mM NaCl group, 70 mM NaCl group and 35 mM NaCl group). *P < 0.05, comparison of rightward circulation among the three groups.

In the Ca^2+^ adsorption experiment, only the NaCl concentration in the extracellular fluid was reduced, and the CaCl_2_ (2.6 mM) and KCl (5 mM) concentrations were unchanged. When the extracellular fluid was changed from set 1 solution (140 mM NaCl) to set 2 solution (105 mM NaCl), after the cells were suspended in the solution for 15 min, the Na^+^ concentration in the solution increased to 106.62 ± 0.61 mM; meanwhile, the Ca^2+^ concentration decreased to 2.59 ± 0.15 mM. When the extracellular fluid was changed from set 1 solution to set 3 solution (70 mM NaCl), the Na^+^ concentration in the solution increased to 73.91 ± 1.05 mM on average after 15 min; the Ca^2+^ concentration decreased to 2.58 ± 0.03 mM on average. The extracellular fluid was changed from set 1 solution to set 4 solutions (35 mM NaCl). After 15 min, the Na^+^ concentration in the solution increased to 41.06 ± 1.75 mM on average; the Ca^2+^ concentration decreased to 2.55 ± 0.01 mM on average. Among them, when the NaCl concentration was changed from 140 to 105 mM and 70 mM, it could be seen that the Ca^2+^ concentration increased by 0.01 mM (Fig. 1a,b).

In the Na^+^ adsorption experiment, only the concentration of NaCl in the extracellular fluid was increased. After set 2, set 3 and set 4 (105 mM, 70 mM and 35 mM NaCl) of solutions were changed to set 1 (140 mM NaCl) solution, after the cells were suspended in the solution for 15 min, the Na^+^ concentration in the solutions of the three groups decreased to 138.34 ± 0.82 mM, 137.41 ± 1.48 mM and 135.64 ± 1.02 mM, respectively; the Ca^2+^ concentration increased to 2.64 ± 0.02 mM, 2.64 ± 0.02 mM and 2.63 ± 0.03 mM, respectively. Among these, when set 2 and set 3 solutions were replaced with set 1 solutions, the Ca^2+^ concentration of minority cases decreased by 0.01 mM (Fig. 1c,d).

In the Ca^2+^ and Na^+^ adsorption experiments, the variation range of K^+^ was 0.032 ± 0.026 mM. Except for a few cases with no change, the rest showed elevated changes, and there was no difference among the groups.

### Changes in ***I***_Na_ and ***I***_Ca-L_ of cells

**Figure 2:**
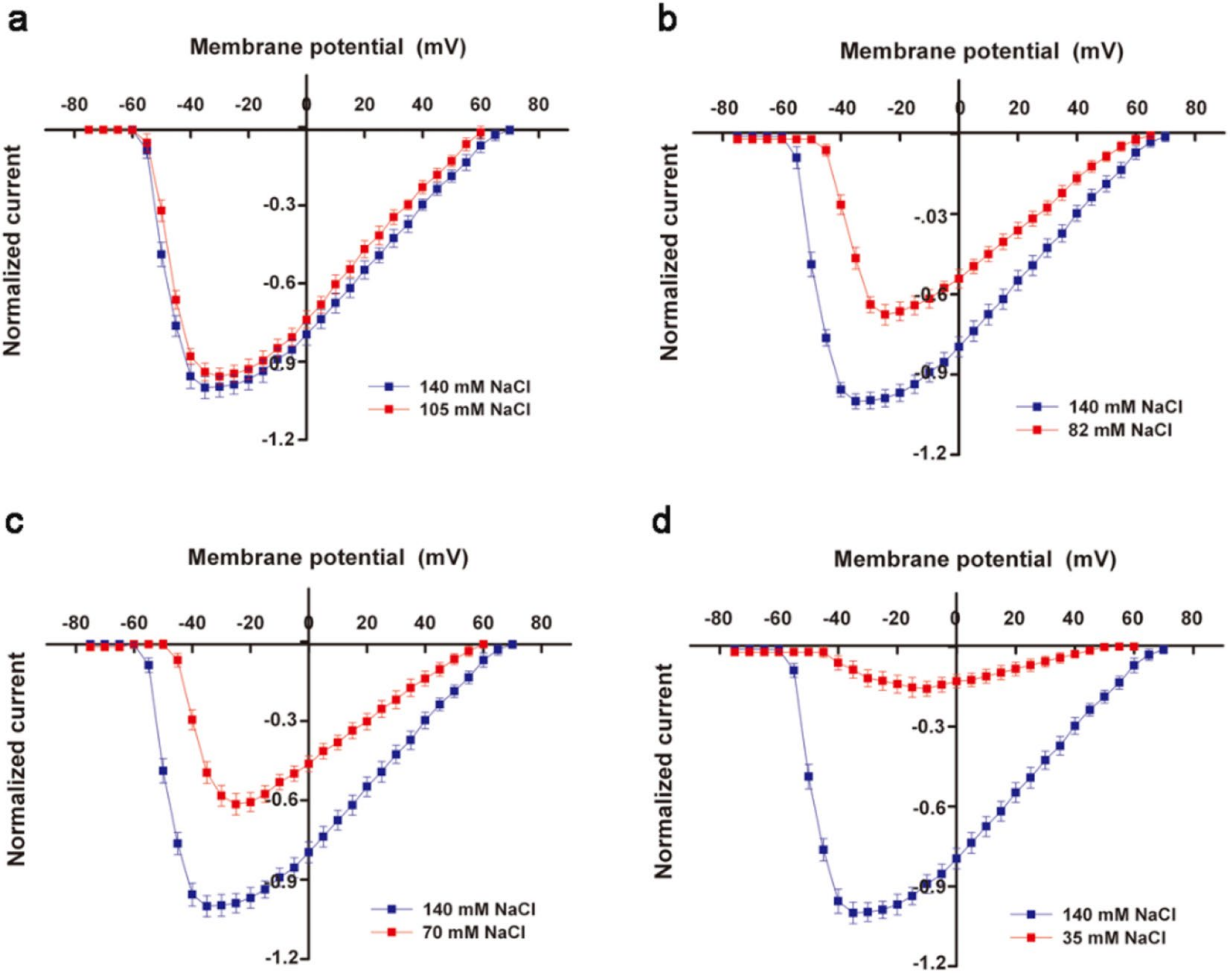
Impact of Na^+^ concentration change in extracellular fluid on normalized *I*_Na_ I/V curve of cardiomyocytes. When the NaCl concentration in the extracellular fluid decreases from 140 to 105 mM, 82 mM, 70 mM and 35 mM, the normalized *I*_Na_ I/V curve changes as shown in the figure. The peak values of 82 mM, 70 mM and 35 mM NaCl groups were compared with the control group, *P* < 0.0001.

As the concentration of NaCl in the extracellular fluid sequentially decreased from 140 to 105 mM, 82 mM (additional point), 70 mM and 35 mM, the normalized I / V curve of *I*_Na_ showed that the peak value (reverse current) gradually decreased, and each group was: 105 mM NaCl group -0.94; 82 mM NaCl group -0.676; 70 mM NaCl group -0.614; 35 mM NaCl group -0.158. Except for the 105 mM NaCl group, there were very significant differences among the other groups and the control group. At the same time, the 82 mM, 70 mM and 35 mM groups showed an increase in the initial potential (activation potential) and peak potential of the I/V curve, and a decrease in the end potential (Fig. 2).

**Figure 3:**
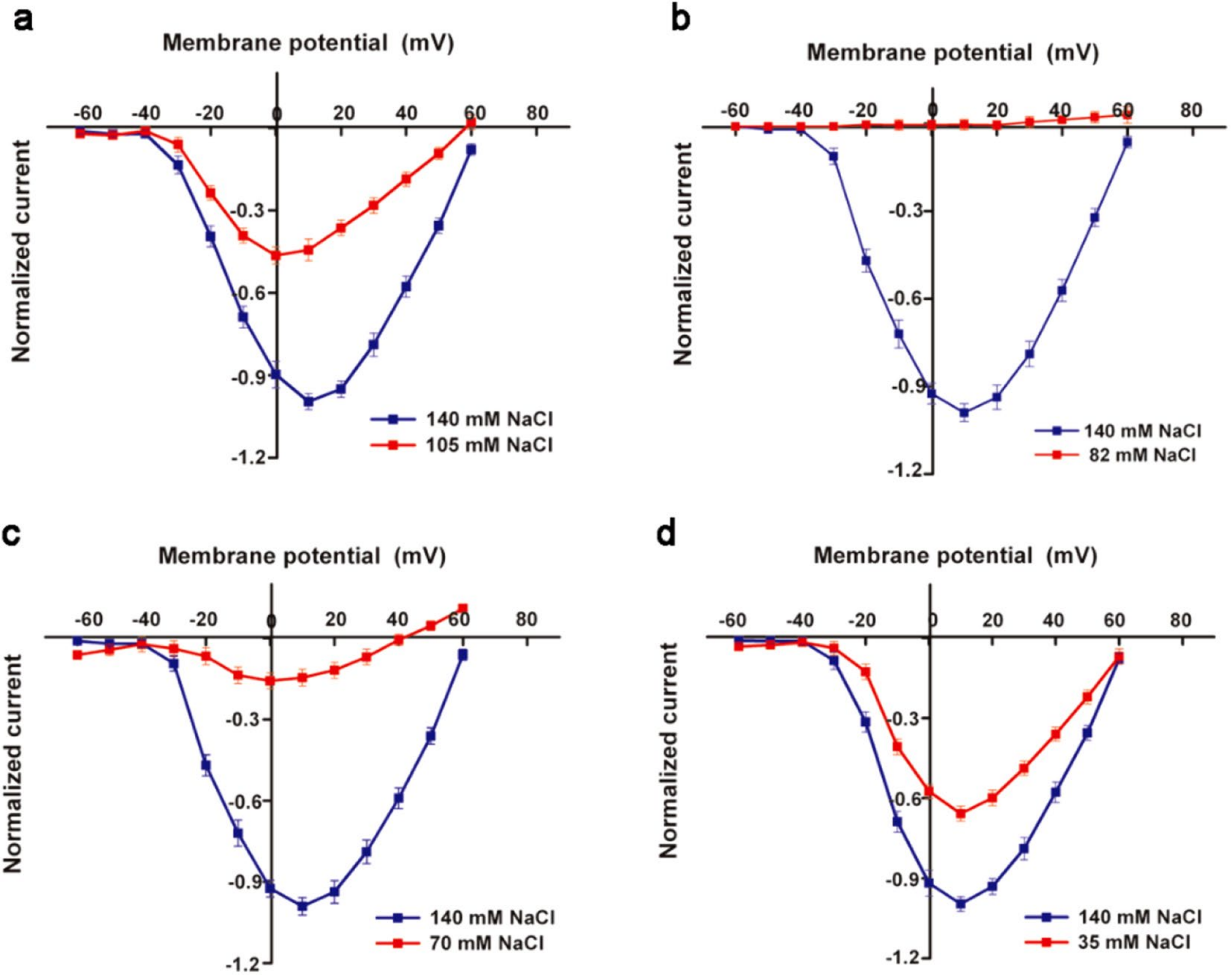
Impact of Na^+^ concentration change in extracellular fluid on normalized *I*_Ca-L_ of cardiomyocytes. When the NaCl concentration in the extracellular fluid decreases from 140 to 105 mM, 82 mM, 70 mM and 35 mM, the normalized *I*_Ca-L_ I/V curve changes as shown in the figure. Among them, the peak value of each group in the experiment was compared with the control group, *P* < 0.0001.

When the NaCl concentration in the extracellular fluid gradually decreased, the peak change of the *I*_Ca-L_ normalized I/V curve showed: -0.464 for 105 mM NaCl group; 0 for 82 mM NaCl group (additional point); -0.158 for 70 mM NaCl group; -0.657 for 35 mM NaCl group. There was a very significant difference between the experimental group and the control group in the comparison of the peak value of the I/V curve. At the same time, the direction of termination current reversed in the 105 mM group and the 70 mM group (Fig. 3).

### Changes in AP of cells

**Figure 4:**
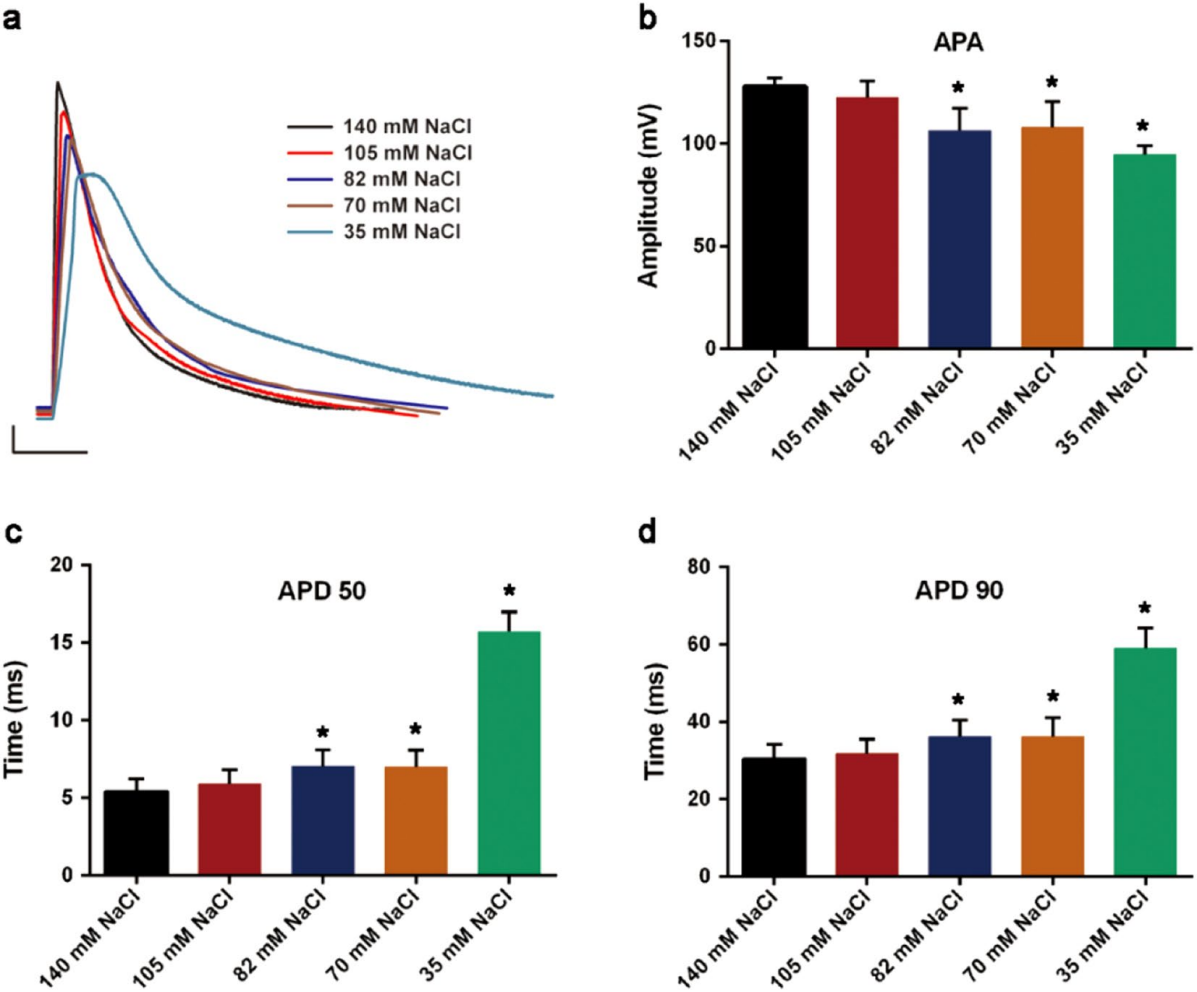
Impact of Na^+^ concentration change in extracellular fluid on AP of cardiomyocytes. When the NaCl concentration in the extracellular fluid decreases from 140 to 105 mM, 82 mM, 70 mM and 35 mM, the changes of AP, APA, APD 50 and APD 90 were shown in the figure (*P < 0.05, compared with the control group). In addition, the APD 50 and APD 90 of the 35 mM NaCl group were compared with the other groups, *P* ≤ 0.0001.

With the gradual decrease of Na^+^ concentration in the extracellular fluid, the change in resting membrane potential (RMP) was -73.06 ± 2.22 mV in the control group; -76.39 ± 2.35 mV in the 105 mM NaCl group; -75.6 ± 2.5 mV in the 82 mM NaCl group; -70.42 ± 6.26 mV in the 70 mM NaCl group; -77.75 ± 1.64 mV in the 35 mM NaCl group. The 105 mM 82 mM and 35 mM NaCl groups were significantly different from the control group (Fig. 4a). The gradual decrease of Na concentration in the extracellular fluid caused the amplitude of action potential (APA) to gradually decrease. The control group was 127.72 ± 4.19 mV; the 105 mM NaCl group was 122.36 ± 8.1 mV; the 82 mM NaCl group was 106.27 ± 11.09 mV; the 70 mM NaCl group was 107.69 ± 12.75 mV; the 35 mM NaCl group was 94.42 ± 4.25 mV (Fig. 4b). In the four experimental groups (105 mM, 82 mM, 70 mM and 35 mM NaCl), the average rates of APA decline were 0.042, 0.168, 0.157 and 0.261, respectively. At the same time, as the Na^+^ concentration in the extracellular fluid decreased, the duration of the action potential (APD) gradually increased. The control group with AP duration at 50% repolarization (APD 50) was 5.41 ± 0.78 ms. The 105 mM NaCl group was 5.86 ± 0.95 ms; the 82 mM NaCl group was 6.99 ± 1.12; the 70 mM NaCl group was 6.97 ± 1.09 ms; the 35 mM NaCl group was 15.7 ± 1.28 ms (Fig. 4c). The control group with AP duration at 90% repolarization (APD 90) was 30.38 ± 3.78 ms, and 31.78 ± 3.64 ms for the 105 mM NaCl group; 36.11 ± 4.27 for the 82 mM NaCl group; 36.08 ± 4.97 ms for the 70 mM NaCl group; 58.99 ± 5.25 ms for the 35 mM NaCl group (Fig. 4d). The extension of APD 50 and APD 90 in the 35 mM NaCl group was very significant, and *P* ≤ 0.0001 compared with other groups.

## Discussion

The understanding of the potential of the surface of biological cells by humans has lasted for more than half a century^13,14^. With the development of molecular biology, the basic composition of biological cell membranes, the role of each component of the membrane in the cell surface potential, the differences in the composition of cell membranes between different organisms, and the physiological functions of membrane components have been known. For example, phosphatidylserine and sialic acid are the main sources of negative charges on the phospholipid membrane and surface glycoconjugates; Na^+^- Ca^2+^ exchangers (proteins) at the plasma membrane level (layer) participate in the completion of cellular functions and electrical activities; and reduced surface sialic acid content inhibits the electrical expression of the plasma membrane^15–22^, or increase or decrease the cell surface negative potential (electric charge) by amphoterics^23,24^, all will affect inward *I*_Na_ and / or *I*_Ca_, and amplitude (APA) time course (APD) of action potential, and even affect cell function. Afterwards, it is discovered that the transmembrane potential participates in the composition of the surface potential of myocardial cells, which makes electrokinetic phenomenon of the cardiomyocyte more complicated^5^, and so on. Undoubtedly, there is a potential on the surface of the biological cell membrane, which is directly related to inflow of Na^+^ and Ca^2+^ , and the relationship between the surface potential of cardiomyocytes and inflow of Na^+^ and Ca^2+^ is more complicated. The difference in the potential composition (components and structure) of the membrane surface is the requirement of different biological cell functions^25,26^.

The fact that the surface potential of cardiomyocytes is composed of two kinds of charges (membrane charges and intracellular charges) shows that the surface of cardiomyocytes has a dense negative charge, and the same amount of positive ions with opposite polarities adsorbed^27^. Among them, one of the important functions of the transmembrane potential is to affect the electrical activity by adsorbing ions on the cell surface (for the channel and ion flow direction)^6^. In the mixed solution (extracellular fluid), the types of ions adsorbed on the surface of the cells are the same as those in the extracellular fluid, but the ratio between ions is different. The total number of adsorbed ions is related to charge (isocharged ion adsorption) and is quantitative (different from extracellular fluid in the body); the amount of each ion adsorbed is mainly determined by the proportion of the ion in the extracellular fluid and the adsorption of the ion the coefficient determines. If only the concentration of one ion in the extracellular fluid is reduced, it is equivalent to increasing the concentration of other ions at the same time; in addition, there are differences in the diffusion strength of monovalent and divalent ions. Therefore, the diffusion of adsorbed ions on the cell surface is different from Fick's law^28,29^.

Theoretically, during the process of depolarization of cardiomyocytes, Na^+^ and Ca^2+^ in the extracellular fluid enter the cell, and there must be no cell surface potential. However, this is not possible: 1. The different ionic composition of the internal and external fluids of the cells, and the way of the ions transmembrane diffusion, directly lead to changes in cell surface potential (appearance and change of transmembrane potential) in electrical activity; 2. If there is no cell surface potential, there is no way to explain the process of opening the ion channel through the induced electric field change of the cell membrane surface structure. Of course, in fact, the cell surface potential exists. Therefore, this imaginary model does not exist. However, under the hypothesis that inflow of Na^+^ and Ca^2+^ in electrical activity are derived from the adsorbed ions on the cell surface, if we gradually reduce the concentration of Na^+^ in the extracellular fluid, the adsorbed ions on the cell surface should be consistent with the changes of the adsorbed ions on cardiomyocyte surface; the changes of inflow *I*_Na_, *I*_Ca-L_ and AP should be consistent with the exchange and diffusion of adsorbed ions on cardiomyocyte surface.

### Characteristics of biofilms

First of all, the ion adsorption of biofilms is different from that of ordinary colloidal particles, and the structure of the charge carrier (charged body) is different, which makes their affinity for different ions very different^30,31^. The affinity of biological phospholipid membranes for Ca^2+^ , Mg^2+^ , Na^+^ and K^+^ is basically the same. The composition of non-phospholipid components in different biology cell membranes makes the overall biological membranes have different affinity for Ca^2+^ , Mg^2+^ , Na^+^ and K^+^. Here, the absorption coefficients of Ca^2+^ , Na^+^ and K^+^ of the phospholipid bilayer, 35, 0.8 and 0.2 M^−1^ were used as a reference^32–34^.

Secondly there is ion exchange of membrane. When the concentration of the main ion Na in the extracellular fluid is gradually reduced, a new proportional relationship is formed between the ions in the extracellular fluid; the adsorbed ions on the membrane then undergo an equal-charge ion exchange according to the new proportional relationship and adsorption coefficient, i.e., some Na^+^ is desorbed, and equally charged Ca^2+^ and K^+^ are adsorbed. Due to the fact that valence of each ion in the extracellular fluid is different (the adsorption rate is different), therefore, ion exchange includes the change of the amount of each adsorbed ion^35–37^, and the exchange changes their position in the ion adsorption layer (and the change of the entire ion adsorption layer shape). That is, “high valence” Ca^2+^ in the deep adsorption layer increases, and some “low valence” Na^+^ is exchanged from the deep adsorption layer to the shallow layer^38–40^.

Furthermore, the diffusion of adsorbed ions is also driven by the electrochemical potential inside and outside the cell. Unlike the diffusion of ions in extracellular fluid, the diffusion of adsorbed positive ions must get rid of (overcome) the attraction of the negative charge (negative potential) on the membrane surface. That is to say, the ions of the adsorption ion layer are divided into two parts: diffusible ions and non-diffusible ions (retaining some ions of tightly adsorption), and the number of diffusible ions determines the amount of inflow ions. Among them, monovalent ions are easier to diffuse than divalent ions; ions located on the surface of the ion adsorption layer are easier to diffuse than ions located in the deep layer^41,42^. With the increase of Ca^2+^ in the ion adsorption layer, the number of diffusible ions will decrease^43–45^; the proportion of Na^+^ and Ca^2+^ in the diffusible ions will also change due to the change in the proportion of Na^+^ and Ca^2+^ in the ion adsorption layer.

### Characteristics of colloid on cardiomyocytes

Cells with different functions have different surface potentials and ion adsorption layers. We have referred to the method of the general adsorption experiment and designed the Ca^2+^ adsorption experiment and Na^+^ adsorption experiment of the cardiomyocytes surface^46^, in which Na^+^ adsorption experiment is the reduction of Ca^2+^ adsorption experiment. The composition of the experimental solution was as close as possible to the extracellular fluid of the patch clamp experiment. The Ca^2+^ adsorption experiment uses 140 mM NaCl as the background solution, and 105 mM, 70 mM, and 35 mM NaCl as the experimental solutions, respectively. Due to the fact that adsorption coefficient of monovalent K^+^ and its proportion in solution ions were very low, the change in the experiment was small, and it was difficult to distinguish it from the intracellular K^+^ release caused by trace cell death in the experiment, K^+^ analysis was omitted from the discussion.

The experimental results show that ion adsorption on the surface of cardiomyocytes, in addition to physical adsorption in general, also has a special potential adsorption. That is, in the Ca^2+^ adsorption experiment results, in addition to the two-way equal charge (adsorption and desorption) positive ion exchange, the unidirectional ion desorption can also be seen. Excluding the overlap of the results of the two adsorption methods, the amount of desorbed Na^+^ is also far greater than twice the amount of adsorbed Ca^2+^ (tens times), and desorption also includes a small amount of divalent Ca^2+^ (in the 105 mM and 70 mM NaCl groups, the Ca^2+^ concentration can be seen to rise). The Na^+^ adsorption experiment can reduce the results (general ion adsorption coefficients and desorption coefficients are slightly different)^30,47^. In both exchange and adsorption, equal-charge ion exchange occurs in the membrane charge, and the amount of exchange is related to the difference between the amount of ions adsorbed by the cells in the two solutions before and after the exchange. Special potential adsorption (or desorption) is a unique ion adsorption (or desorption) mode of the cardiomyocytes surface. It is related to the spatial effect of transmembrane charge and is determined by the mode of cardiomyocytes repolarization. The amount of desorbed ions is proportional to the difference between the concentration of the two extracellular fluids before and after exchange; the type of desorbed ions is related to the distribution of adsorbed ions; desorption begins on the outer surface (related to electric field force). There is no doubt that the Na^+^ concentration is gradually reduced, the difference in the ratio of Na^+^ and Ca^2+^ in the adsorption ion layer is further reduced by the special potential adsorption, and the amount of Ca^2+^ is further increased. This may result in changes in the distribution of adsorbed ions^48^ (exchange of Na^+^ and Ca^2+^ positions, the increase of Ca^2+^ adsorbed by the phospholipid layer) and the shape of the adsorption ion layer (decreased diffusible ions)^49^.

Briefly describe the repolarization process of cardiomyocytes: the electrical activity of cardiomyocytes in vivo is initiated by external electric field and complete in the external electric field. If the influx ions originate from the adsorbed ions on the cell surface, then the outflow of repolarized intracellular ions (including plasma membrane ion exchange) can only return to the adsorbed layer on the cell surface. And the inflow Na^+^ and Ca^2+^ have only one way to return, namely the ion exchange of the plasma membrane. This includes two ways: direct 3Na^+^-Ca^2+^ exchange (positive direction), Ca^2+^ is returned; and through the channel opening to the surface glycoconjugates layer, K^+^ flows out to the adsorption layer, and then diffuses to the plasma membrane layer, and then undergoes 3Na^+^-2 K^+^ exchange, Na^+^ is returned. The return process of the adsorbed ions mainly has the following characteristics: 1. there is a distance of 50 nm between the surface glycoconjugates and the plasma membrane^50^, and the two positive ion flow directions formed in two ways are “opposite” (K^+^ outflow and 3Na^+^-2 K^+^ exchange); 2. K^+^ outflow and 3Na^+^-2 K^+^ exchange are accompanied by the generation of negative transmembrane potential; 3. At the same time, the local dynamic electrochemical balance of the adsorption layer is accompanied; 4. The completion of plasma membrane ion exchange lags behind the channel K^+^ outflow. The combined effect of several aspects constitutes the special relationship among membrane negative charge, negative transmembrane potential and adsorbed ions; it makes the distribution of adsorbed ions on the cell surface and the shape of the ion adsorption layer unique. Its electrokinetic characteristics are completely different from ordinary colloidal particles. When the transmembrane potential changes, the change of mobility is repeated oscillation within a certain range (Supplementary Fig. 1); when the ion concentration changes, high ion concentration companies high mobility while low ion concentration companies low mobility (Supplementary Fig. 2). These are consistent with the results of special electric potential adsorption (or desorption) in the cardiomyocyte adsorption experiments.

The characteristics of ion adsorption and exchange on the surface of intact live cardiomyocytes reveal that the internal and external fluids of the cardiomyocytes, the adsorption ion layers of inside and outside the membrane, are connected together by the transmembrane potential. When the change of a certain component breaks the original electrochemical balance, a new electrochemical balance is achieved through local linkage adjustment. Re-examine the electrical activity of cardiomyocytes, which is essentially a circular cycle process (inside and outside the cell) driven by a dynamic electrochemical potential.

### Analysis of changes in ***I***_Na_, ***I***_Ca-L_ and AP

When the NaCl concentration in the extracellular fluid was reduced from 140 to 105 mM, 82 mM, 70 mM and 35 mM, the peak value of the *I*_Na_ normalized I / V curve sequentially decreased. The rate of decline varies, starting small and finally large. The 82 mM, 70 mM and 35 mM NaCl groups showed that the activation potential and peak potential increased, and the end potential decreased. And the peak change trend of the *I*_Ca-L_ normalized I/V curve was that the 105 mM group decreased; the 82 mM group was zero; and the 70 mM and 35 mM groups increased. The 82 mM group (zero for peak) was the reversal point of the normalized I/V curve peak change of the "V" type *I*_Ca-L_ recorded along the peak change trend by refining experimental conditions. In addition, in 105 mM, 82 mM and 70 mM NaCl groups, the flow of ions at the end of the I/V curve was reversed.

During the gradual reduction of the concentration of Na^+^, there were two kinds of responses, such as equal charge exchange and simple ions desorption, on the adsorption ion layer of the cardiomyocytes surface. After the Na^+^ concentration was lowered, the Ca^2+^ content in the surface adsorption ions gradually increased. In the early stage (105 mM NaCl), it mainly lost Na^+^, narrowed the ratio difference between Ca^2+^ and Na^+^ in the adsorbed ions, and exchanged the location of Ca^2+^ and Na^+^ in the ion adsorption layer. Some Ca^2+^ changes from diffusible ions to non-diffusible ions in exchange for Na^+^ from non-diffusible ions to diffusible ions. After the exchange, the peak value of the *I*_Na_ I/V curve decreased only slightly (about 6%); and the peak value of the *I*_Ca-L_ I/V curve decreased. In the middle-late period (82–35 mM NaCl), with the increase of ion desorption (Na^+^ dominated), Ca^2+^ in the adsorption ion layer also increased further. When the ratio of Ca^2+^ and Na^+^ in non-diffusible ions was close to the extreme value, the exchange of positions became slow, and the proportion of Ca^2+^ in diffusible ions started to increase. After the exchange was met, the peak value decline rate of the *I*_Na_ I/V curve gradually increased (32.4% to 84.2%). The peak value of the *I*_Ca-L_ I/V curve changed from decrease to occurrence of the reversal point to rising. The decrease in the amount of Na^+^ in the diffusible ions; the increase in the amount of Ca^2+^ in the whole adsorbed ions and the change in the shape of the adsorption ion layer were the reasons for the changes in the activation potential, peak potential and end potential of the *I*_Na_ I/V curve. At the end of the I/V curve, the flow of ions was reversed, because in the patch clamp experiment, the applied intracellular stimulus pulse (amplitude of the transmembrane potential) was too high and exceeds the equilibrium point of the electrochemical potential inside and outside the cell. It should be recorded that the ion flow at the end of the I/V curve was reversed. Of course, the reversal of ion flow was also related to the number of ions at the level of the extracellular ion adsorption layer corresponding to the intensity of the voltage applied inside the cell.

The results of the action potential showed that 1. APA gradually decreased, the rate of decrease was much smaller than the rate of Na^+^ decrease in the extracellular fluid; it was more in line with the two reasons of desorption of adsorbed ions on the cell surface and increase of Ca^2+^ ratio in the ion adsorption layer. The number of diffusible ions was reduced. 2. APD 50 and APD 90 were gradually prolonged. When the concentration of Na^+^ in the extracellular fluid dropped to 105–70 mM, *I*_Ca-L_ decreases, or rose slightly after reversal (inflow Ca^2+^ decreases), and *I*_Na_ gradually decreases (inflow Na^+^ gradually decreases); and the adsorbed Ca^2+^ in the deep layer of adsorbed ions (plasma membrane layer) gradually increased, adsorbed Na^+^ gradually decrease. On the one hand, the reverse mode of Na^+^-Ca^2+^ exchange was turned on, which gradually increased the load of Na^+^ and Ca^2+^ exchange. On the other hand, the reduction of Inflow Na^+^ and plasma membrane adsorption Na^+^ had caused the shortage of Na as the raw material for Na^+^-Ca^2+^ exchange and Na^+^-K^+^ exchange, which will undoubtedly prolong the APD (APD 50 and APD 90). Of course, the effect of acute high glucose on the prolongation of APD cannot be completely ruled out here. However, in the adsorption experiment, the change of K^+^ suggests that high sugar is not the main reason for the prolongation of APD. When the concentration of Na^+^ in the extracellular fluid decreased to 35 mM, *I*_Na_ decreased significantly (Inflow Na^+^ decreased significantly); *I*_Ca-L_ increased significantly after "V" type reversal; on the plasma membrane layer, the adsorbed Ca^2+^ increases further, and the adsorbed Na^+^ decreases further. Na^+^-Ca^2+^ reverse exchange still existed, Na^+^ was lacking inside and outside the membrane, the time for Na^+^-Ca^2+^ exchange and Na^+^-K^+^ exchange was further increased, and ion exchange was seriously overloaded. The shape of the action potential and APD were significantly changed. In addition, there was a significant difference in RMP between the 105 mM, 82 mM, and 35 mM NaCl groups compared with the control group. The overall and pair-wise data analysis considered that the difference was not significantly related to the reduction of Na^+^ concentration in the extracellular fluid.

In the 1990s, research of Post and Ji for amphiphiles^51,52^ evidenced our hypothesis: they applied amphiphiles to cardiomyocytes and found that increasing or decreasing the negative potential on the cell surface could increase or decrease *I*_Na_ and *I*_Ca-L_. This method was to implant some ion-adsorbing carriers (positive charge or negative charge) on the cell surface, and then increased or reduced the number of positive ions adsorbed; directly demonstrated the relationship among influent ions and the cell surface potential, and adsorbed ions on the surface. Due to the placement of the amphiphile they chose was limited to the phospholipid membrane, therefore, the distribution of the implanted charge was also uneven. Affected by the characteristics of ion adsorption, there are not only changes in the amount of ions adsorbed on the cell surface, but also changes in distribution (some adsorbed Ca^2+^ is transferred or exchanged from the shallow layer to the deep layer). Affected by the characteristics of ion adsorption, there are not only changes in the amount of ions adsorbed on the cell surface, but also changes in position (distribution changes). As the articles results show, the changes of *I*_Ca-T_, APD and ion current, etc. In addition, about the relationship between amphiphiles and ion channels mentioned in the article, our research shows that the combined action of the surface potential components of cardiomyocytes can regulate the opening of ion channels, but it requires the help of adsorbed ions. The increase or decrease of the surface potential can cause a positive correlation change of influent ions because the surface potential is an equal charge carrier that adsorbs ions; while influent ions originate from the surface adsorption ions. Regarding the relationship between surface potential and ion channel, we have introduced it in another article.

The above experimental results were consistent with the rule of exchange and diffusion of adsorbed ions on the cardiomyocytes surface. The influx ions originated from the adsorbed ions on the cell surface, and also coincided with the relevant details during the electrical activity such as the number of diffusible adsorbed ions and the inactivation of the inflowing ion current; the distribution of the adsorbed ions and the sequence of the inflow of ions; after the influx of ions, the change of the ion adsorption layer and the ion exchange of the plasma membrane (exchange requires special environment) and the formation of the depolarization potential; and so on. These are the result of the diffusion of ions in the solution (extracellular fluid) and the effect of high glucose, which cannot be replicated. Therefore, it can be determined that the hypothesis holds. In the process of depolarization of cardiomyocytes, influent ions are derived from adsorbed ions on the cell surface.

### In-depth understanding

When we continue the above understanding and outline the curve of the surface potential change of cardiomyocytes during electrical activity, it is easy to obtain the mechanism of electrocardiogram generation: cardiomyocytes encounter an external electric field (from sinus node or adjacent charged cells or pacemaker) → the surface lost adsorbed ions and becomes charged (separation of the electric double layer on the cell surface), and the ion channel is opened → the adsorbed ions on the cell surface flow into the cell (Na^+^ and Ca^2+^), the end of depolarization → the cell surface loses more adsorbed positive ions, forming a large potential difference on the cell surface (i.e. depolarization potential); At the beginning of repolarization, intracellular K^+^ outflow and ion exchange on the plasma membrane, The outflow ions stop at the adsorption layer on the cell surface → the negative potential on the cell surface gradually decreases, and the cell repolarization ends (the transmembrane potential returns to the resting potential level), but the repolarization of the entire heart does not end (electric double layer remains in a separated state) → there is still a potential difference on the cells surface (with a Zeta potential), which was the repolarization potential. The large depolarization potential is the basis of QRS wave formation in electrocardiogram, and the small repolarization potential was the basis of T wave formation. Each cardiomyocyte has a pacemaker effect (charged) in the process of electrical activity, and conducts in the manner of field transmission (electric field propagation). The field strength affects the conduction speed of electrical activity between cells. Whether a cardiomyocyte has autorhythmicity depends on its surface potential structure.

Re-understand the electrical activity process of cardiomyocytes, and find that during the entire electrical activity, the cardiomyocytes are in a relatively 'isolated' space in the external electric field (surface electric double layer separation). This causes a fact that the starting point of inflow ions and the end point of outflow ions are the same; and then, the adsorbed ions species inside and outside the membrane are exchanged. Due to the dynamic changes of the two ends of the electrochemical potential balance and the structure of the adsorption ion layer in the whole process; and the ion composition of the adsorption ion layer obeys the surrounding ion conditions, it is not difficult to find that the active diffusion (transport) of ions does not exist, at least in the electrical activity of cardiomyocytes.

In this study, we strive to fully consider the integrity of cardiomyocyte structure and the relationship between components; comply with the colloidal properties of cardiomyocytes, and show the characteristics of adsorption, exchange and diffusion of adsorbed ions on the cardiomyocyte surface. For example, we select living cells (reserve transmembrane potential) as experimental materials; reject the use of blockers and fluorescent stains to avoid the effects of drugs and reagents on membrane charge; the concentration of Na^+^ and total ion in extracellular fluid should be reduced simultaneously. The theoretical reasoning conforms to the basic principles of physical chemistry and the characteristics of biological cells, and is consistent with the relevant scientific experiment ‘results’. The inflow of ions is only a link of the process in electrical activity. The addition of new knowledge should ensure the coherence and integrity of the entire circulation process driven by the electrochemical potential inside and outside the cell (includes large whole, small parts; total electrochemical potential, single ion; and dynamic changes in structure, etc.). We hope that the research results can expand the thinking of biological research and promote the development of cardiovascular basic and clinical research.

## Materials and methods

### Cell preparation

Ventricular cardiac myocytes were isolated from adult Sprague–Dawley rats (2–3 months old, weight 225–300 g) using standard enzymatic techniques, as described previously^53^. Freshly isolated single cells were stored in Tyrode’s solution containing 137 mM NaCl, 5.4 mM KCl, 1.2 mM MgCl_2_, 1 mM NaH_2_PO_4_, 1 mM CaCl_2_, 20 mM glucose, and 20 mM HEPES (pH 7.4).

All animal protocols were consistent with current United States National Institutes of Health guidelines, and all of the studies were approved by the Committee on the Use and Care of Animals of the Eighth Medical Center of the People's Liberation Army General Hospital. And the research also followed the requirements of ARRIVE guidelines 2.0.

### Ions adsorption studies

#### Preparation of solution

The ion adsorption experiment includes Ca^2+^ adsorption experiment and Na^+^ adsorption experiment. The two experiments included three identical groups: 105 mM NaCl group, 70 mM NaCl group, and 35 mM NaCl group; each shared 4 sets of solutions; only their background solutions and experimental solutions were selected differently^54^. The composition of the solution set 1 was 140 mM NaCl, 2.6 mM CaCl_2_, 5 mM KCl, 10 mM glucose. On the basis of set 1 solution, sets 2–4 solutions had reduced NaCl concentration by 25%, 50%, 75%, and correspondingly the concentration of glucose was increased to achieve physiological osmotic pressure. The composition of the solution set 2 was 105 mM NaCl, 2.6 mM CaCl_2_, 5 mM KCl, 76 mM glucose; set 3, 70 mM NaCl, 2.6 mM CaCl_2_, 5 mM KCl, 152 mM glucose, and set 4, 35 mM NaCl, 2.6 mM CaCl_2_, 5 mM KCl, 228 mM glucose (Imitated extracellular fluid of patch clamp experiment), respectively. Among them, the CaCl_2_ concentration was chosen to be 2.6 mM in order to reduce the cell death rate during the experiment. For Ca^2+^ adsorption experiment, set 1 solution was used as the background solution of three groups; and sets 2–4 solutions were used as experimental solutions of 105 mM, 70 mM, and 35 mM NaCl groups, respectively. For the Na^+^ desorption experiment, sets 2–4 solutions were used as the background solutions of 105 mM, 70 mM, and 35 mM NaCl groups, and set 1 solution was used as the experimental solution of the three groups.

#### Experiment process

The cells were divided into 6 groups, of which Ca^2+^ adsorption and Na^+^ adsorption experiments had 3 groups. Each group of cells was 200 μl (after centrifugation; about 158,000). Each group was rinsed twice with their respective background solutions, with an interval of 10 min each time, during which the cells were kept in suspension. Before the end of the second rinse in each group, 10 μl of suspension was taken for cell counting and for checking the cell status (only cells had survival rate ≥ 80% were used for experiment). Cells were centrifuged (500 rpm * 3 min) to change the experimental solution. 800 μl of experimental solution was added to each group. In Ca^2+^ adsorption experiment, sets 2, 3 and 4 of solutions were added to cells respectively. In Na^+^ adsorption experiment, set 1 solution was added. The cells were kept in suspension for 15 min, and cells were counted and the status of the cells was checked (the survival rate of the cells must be ≥ 70%). 790 μl of the cell suspension was taken, and the experiment was ended. NexION 2000 ICP-MS (Perkin Elmer) was used to detect the content of Na^+^, K^+^ and Ca^2+^ in the final solution of the experiment, and the experiment was completed once. Experiments that fully met the requirements of the process were repeated 12 times.

### Electrophysiological studies

#### Preparation of solution

Divide the experimental solution into two parts (for recording *I*_Ca-L_ and for recording *I*_Na_ and AP); each part included pipette solution and external solution (extracellular solution). Among them, the extracellular fluid was divided into the control group and the experimental groups that gradually reduced the concentration of NaCl based on the control group (in turn, 25%, 50%, 75%, and an additional point, 41.4%).

For *I*_Ca-L_ recording, the pipette solution contained: 125 mM CsCl, 7.0 mM MgCl_2_, 1.0 mM CaCl_2_, 5.0 mM Na_2_ATP, 11 mM EGTA, 10 mM HEPES (pH 7.2, adjusted with CsOH). The composition of extracellular fluids is shown in Table 1.

**Table 1:** The extracellular fluid used in electrophysiological experiments.

Group	Solutions composition (mM)							
	NaCl	KCl	CaCl_2_	MgCl_2_	NaH_2_PO_4_	Glucose	Hepes	pH *
***I***_**Ca-L**_								
Control group	140	5	3		0.33	10	10	7.35
Experiment-1	105	5	3		0.33	76	10	7.35
Experiment-1 +	82	5	3		0.33	126	10	7.35
Experiment-2	70	5	3		0.33	152	10	7.35
Experiment-3	35	5	3		0.33	228	10	7.35
***I ***_***Na***_** and AP**								
Control group	140	5	3	1		10	10	7.35
Experiment-1	105	5	3	1		76	10	7.35
Experiment-1 +	82	5	3	1		126	10	7.35
Experiment-2	70	5	3	1		152	10	7.35
Experiment-3	35	5	3	1		228	10	7.35

*Adjust pH with NaOH.

For *I*_Na_ and AP recording, the pipette solution contained: 136 mM K-aspartate, 5.4 mM KCl, 1.0 mM MgCl_2_, 5.0 mM EGTA, 5.0 mM, MgATP, 5.0 mM Phosphocreatine (pH 7.2, adjusted with KOH). The composition of extracellular fluids is shown in Table 1.

#### Experiment process

All the whole cell patch clamp recordings were made by single ventricular myocytes using patch clamp amplifier epc-10 and data acquisition software patchmaster (Heka electronic, Lambrecht, Pfalz, Germany) (temperature 24 ± 1 ℃) under the condition of control and successively reducing the concentration of NaCl in extracellular fluid. Almost all experiments were carried out in pairs in different cell groups (by perfusion method). After the initial operation process (preparation, membrane pipette sealing, membrane breaking, capacitance compensation, correction, etc.)^55,56^, the action potential was recorded in the current clamp mode. Record *I*_Ca-L_ and *I*_Na_ in voltage clamp mode.

For the *I*_Ca-L_ recording, hold the voltage at -50 mV for 50 ms, in the test voltage range of -60 to + 60 mV, in steps of 10 mV, with 500 ms depolarization pulses (2 Hz), trigger *I*_Ca-L_. For *I*_Na_ recording, from a holding voltage of -120 mV (100 ms) to a test voltage range of -100 to + 100 mV in 5 mV steps with a pulse duration of 100 ms and a frequency of 0.5 Hz.

Cells (epicardial cell) with similar morphology, similar size, and clear appearance were selected as experimental objects. For each experimental condition (n = 6), no ion blocker was used to avoid affecting the cell surface potential.

### Statistical analyses

Data was expressed as means and standard deviation. Group comparisons of data were analyzed by paired (or unpaired) t-test (GraphPad Prism 6). Values with *P* < 0.05 were considered statistically significant.

## Supplementary Information

Supplementary Information
