# Supplementary material for: New understanding of electrical activity brought by surface potential of cardiomyocytes
Source: Sci Rep. 2021 Mar 23;11:6593. doi: 10.1038/s41598-021-86138-w (PMC7988015; doi:10.1038/s41598-021-86138-w)
Supplement: Supplementary file 1 — Supplementary Information [file 41598_2021_86138_MOESM1_ESM.pdf]

New understanding of electrical activity brought by surface potential of cardiomyocytes

Ying Zhou [*], Yanfei Hao, Pei Sun, Guang Li, Mengqi Dong, Xuehui Fan, Xiuyun He

Supplementary Figure 1

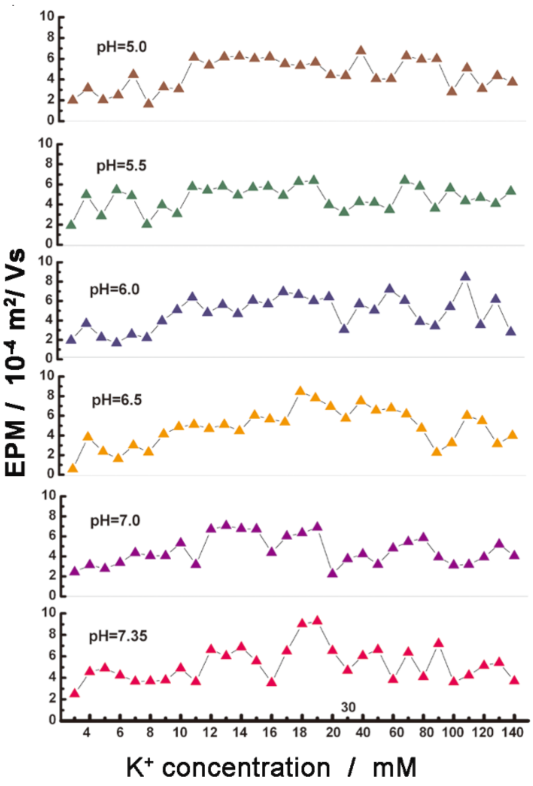

Supplementary Fig. 1 | Plot of electrophoretic mobility for cardiomyocytes against the concentration of K^+ in rang of 3-140 mM. The data were all measured using extracellular fluids at an ionic concentration of 153 mM (147-10 mM NaCl, 3 mM $CaCl_2$) at different pH values. For experimental procedures, see Reference 5.

Supplementary Figure 2

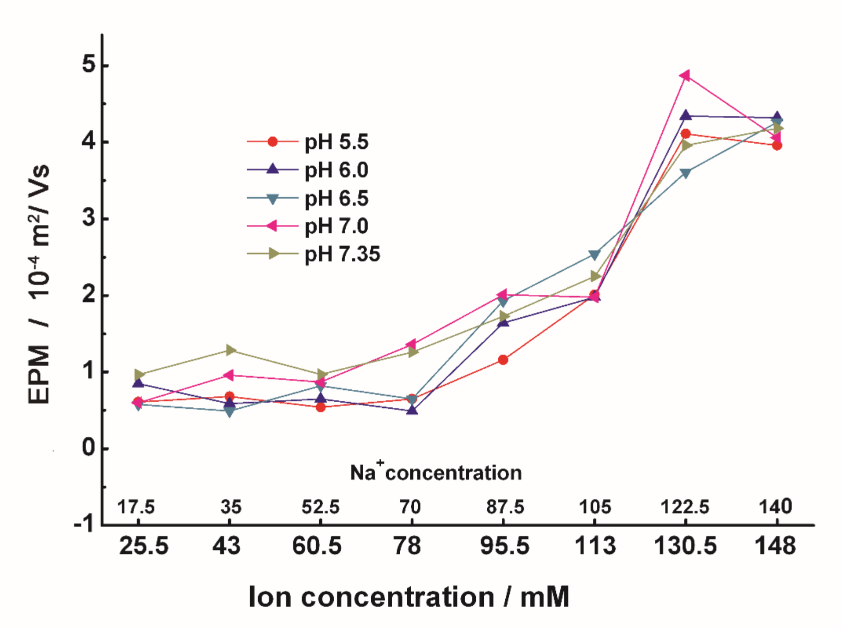

Supplementary Fig. 2 | The fluctuation trend of electrophoretic mobility measured in extracellular fluids with ion concentration of 25.5-148 mM (among them, 5 mM KCl, 3 mM $CaCl_2$, 17.5-140 mM NaCl, 266-10 mM glucose) and pH 7.35. For the experimental procedure, the same as Supplementary Figure 1.
